# Effect of preoperative segmental range of motion on patient outcomes in cervical disc arthroplasty

**DOI:** 10.1186/s12891-020-03419-7

**Published:** 2020-07-13

**Authors:** Ting-kui Wu, Hao Liu, Chen Ding, Xin Rong, Jun-bo He, Kang-kang Huang, Ying Hong, Bei-yu Wang

**Affiliations:** Department of Orthopedic Surgery, West China Hospital, Sichuan University, No. 37 Guo Xue Rd, Chengdu, 610041 Sichuan China

**Keywords:** Cervical disc arthroplasty, Range of motion, Segmental mobility, Limited motion, Excessive motion

## Abstract

**Background:**

Cervical disc arthroplasty (CDA) has been demonstrated, in clinical trials, as an effective and safe treatment for patients diagnosed with radiculopathy and/or myelopathy. However, the current CDA indication criteria, based on the preoperative segmental range of motion (ROM), comprises a wide range of variability. Although the arthroplasty level preserved ROM averages 7°-9° after CDA, there are no clear guidelines on preoperatively limited or excessive ROM at the index level, which could be considered as suitable for CDA.

**Methods:**

This was a retrospective study of patients who underwent CDA between January 2008 and October 2018 using Prestige-LP discs in our hospital. They were divided into the small-ROM (≤5.5°) and the large-ROM (> 12.5°) groups according to preoperatively index-level ROM. Clinical outcomes, including the Japanese Orthopedics Association (JOA), Neck Disability Index (NDI), and Visual Analogue Scale (VAS) scores, were evaluated. Radiological parameters, including cervical lordosis, disc angle (DA), global and segmental ROM, disc height (DH), and complications were measured.

**Results:**

One hundred and twenty six patients, with a total of 132 arthroplasty segments were analyzed. There were 64 patients in the small-ROM and 62 in the large-ROM group. There were more patients diagnosed with cervical spondylosis in the small-ROM than in the large-ROM group (*P* = 0.046). Patients in both groups had significantly improved JOA, NDI, and VAS scores after surgery, but the intergroup difference was not significant. Patients in the small-ROM group had dramatic postoperative increase in cervical lordosis, global and segmental ROM (*P* < 0.001). However, there was a paradoxical postoperative decrease in global and segmental ROM in the large-ROM group postoperatively (*P* < 0.001). Patients in the small-ROM group had lower preoperative DH (*P* = 0.012), and a higher rate of postoperative heterotopic ossification (HO) (*P* = 0.037).

**Conclusion:**

Patients with preoperatively limited segmental ROM had severe HO, and achieved similar postoperative clinical outcomes as patients with preoperatively excessive segmental ROM. Patients with preoperatively limited segmental ROM showed a postoperative increase in segmental mobility, which decreased in patients with preoperatively excessive segmental ROM.

## Introduction

In recent decades, cervical disc arthroplasty (CDA) has been studied in many clinical trials as an alternative surgical treatment to anterior cervical discectomy and fusion (ACDF), due to a paradigm shift towards preserving motion and avoiding adjacent segment disease [1–7]. Segmental range of motion (ROM) has been commonly accepted, in published FDA-approved trials, as an indication for using CDA. These trials suggest that preoperative segmental ROM should range between 2° to 11° or 2° to 20° on lateral flexion-extension X-rays [4–9], presenting patients who have undergone CDA with a wide range of variability in segmental motion. There are no clear guidelines as to the optimal preoperative index-level ROM; although the preoperative ROM at the index level averaged 7°-9°, and a similar motion was successfully preserved after surgery [10–14]. These observations raise a question for surgeons; whether limited or excessive preoperative ROM, other than the average one at the index level, could also achieve satisfactory clinical or kinematic outcomes?

There is limited data to answer the question above. Tu et al. [10] concluded that preoperatively less-mobile patients (ROM ≤5°) had similar clinical improvements, but showed a greater increase in segmental mobility than more-mobile (ROM > 5°) patients. However, some patients in the more-mobile group had excessive segmental ROM that may affect the results. To our knowledge, no clinical study on CDA has specifically analyzed patients with preoperatively excessive ROM at the index level. Patients selection is crucial to guarantee all the benefits of CDA. This study aims to investigate the influence of preoperative index-level ROM on postoperative ROM after CDA, and whether the patients with preoperatively limited or excessive segmental ROM are suitable candidates for arthroplasty.

## Methods

### Patients

Patients (312) who underwent CDA or hybrid surgery (HS) in the West China Hospital of Sichuan University, using Prestige-LP discs between January 2008 and October 2018, were retrospectively reviewed. The surgical indications were intractable symptomatic radiculopathy and/or myelopathy caused by cervical degenerative disc disease (DDD) or spondylosis at 1-or 2-levels, from C3-C7. Exclusion criteria for arthroplasty were: 1) severe facet joint degeneration, 2) ossification of the posterior longitudinal ligament (OPLL), 3) segmental ROM < 2°, 4) segmental instability (> 3.5 mm sagittal plane translation or > 20° sagittal plane angulation), 5) intervertebral disc height loss more than 50% and, 6) less than 12 months follow-up period. ACDF was performed if radiographic signs of instability, bridging osteophytes, and severe facet degeneration were observed in the 2-level disease. The study protocol was approved by the institutional ethics committee, and all patients signed informed consent.

### Cutoff values for preoperative segmental ROM

**Fig. 1:**
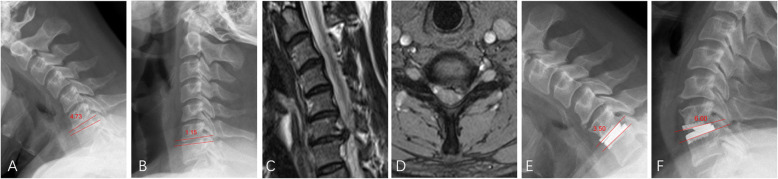
A patient underwent CDA at C6/7 using Prestige-LP discs in the small-ROM group. Preoperative segmental ROM was measured at 3.48° using lateral flexion-extension X-rays (**a** and **b**). Preoperative MRI demonstrated disc herniation at C6/7 (**c** and **d**). X-rays at 50 months follow-up (**e** and **f**) showing increased segmental mobility (ROM = 9.50°) at the arthroplasty segment

**Fig. 2:**
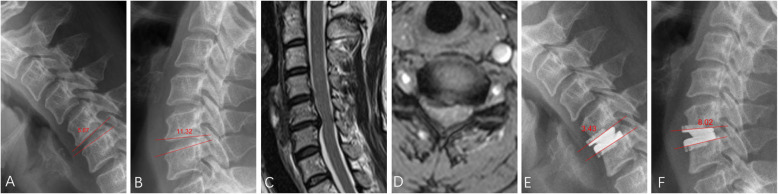
A patient in the large-ROM group underwent CDA at C5/6 using Prestige-LP discs. Preoperative segmental ROM was measured at 17.19° using lateral flexion-extension X-rays (**a** and **b**), and MRI showed disc herniation at C5/6 (**c** and **d**). The X-rays recorded at 50 months follow-up (**e** and **f**) showing decreased segmental mobility (ROM = 11.45°) at the arthroplasty segment

There is no consensus on cutoff values for relatively small or large ROM at the index level for surgery. Tu et al. [10] divided patients based on C5/6 preoperative ROM of ≤5° and > 5°, without explanation. Kang et al. [15] defined cutoff values as 10°, to group patients according to the average segmental ROM at the last follow-up. In the current study, the mean and standard deviation (SD) of radiographic data of the segmental ROM at the index levels, were calculated. Each target disc in 2-level CDA surgery was considered as an independent data point. The data were normally distributed around the mean of 9.01° with an SD of 3.47° (K-S test, *P* = 0.200). Based on the raw data, the cutoff values for preoperative small ROM were defined as mean - SD, while large ROM was mean + SD. Therefore, the small-ROM group was defined as having segmental ROM of ≤5.5° at the index level (Fig. 1) and the large-ROM group as > 12.5° (Fig. 2).

### Surgical techniques

All operations were carried out by the same senior surgeon (H.L.). A standard right-sided anterior cervical approach was performed after general anesthesia. Thorough decompression was done at the index levels by removing the disc tissue, posterior longitudinal ligament, and osteophytes to achieve neural decompression. Appropriated Prestige-LP disc (Medtronic Sofamor Danek, Memphis, Tennessee) or Zero-P implant (Synthes, Oberdorf, Switzerland) was inserted into the intervertebral space after the endplates were well prepared. Then, C-arm fluoroscopy was performed to verify the proper placement of the implants. A drainage tube was inserted after irrigation and hemostasis before the closure of the incision.

### Data collection

The clinical and radiographic outcomes of patients were routinely evaluated at regular intervals: before surgery, at 1 week, 3, 6, 12 months postoperatively, and at the last follow-up. Clinical outcomes were evaluated according to validated self-assessment questionnaires, including the Japanese Orthopedics Association (JOA), Visual Analogue Scale (VAS), and Neck Disability Index (NDI) scores. Radiological parameters including cervical lordosis, disc angle (DA) of the arthroplasty segments, ROM of C2 - C7 and the arthroplasty segments, and disc height (DH) were measured in lateral radiographs in neutral, extension and flexion views. Global and segmental ROMs were defined as the difference in respective Cobb angles between flexion and extension views. We applied the McAfee classification system (Grades 0 to 4) to classify heterotopic ossification (HO) [16]. McAfee grades 0–2 were defined as low-grade HO and grades 3–4 as high-grade HO, based on impaired ROM criteria [17]. Adjacent segment degeneration (ASD) was evaluated based on the narrowing of the disc space and new formation or enlargement of anterior osteophytes on lateral radiographs [18]. Prosthesis subsidence was defined as > 2 mm height loss of anterior or posterior functional spinal unit (FSU) when compared with that of the immediate postoperative radiograph.

### Statistical analysis

Statistical analysis was performed using SPSS software version 19.0 (IBM SPSS Inc., New York, USA). The results were presented as mean  ±  SD or percentages. A paired t-test was used to compare preoperative and postoperative parameters. The independent t-test or the Mann–Whitney U test was used to compare continuous variables between the two groups. The Chi-square or Fisher’s exact test was used for inter-group categorical variables. One-way ANOVA was used for continuous variables among the three surgical types. Tests were two-tailed with *p* < 0.05.

## Results

### Demographic data

One hundred and 26 patients underwent 1-or 2-level surgery for a total of 132 arthroplasty segments (Table 1). Sixty-four (male/female: 32/32) were placed in the small-ROM group, and 62 (male/female: 21/41) in the large-ROM group, with a mean follow-up of 37.12 months. The mean age was 46.11 years in the small-ROM and 43.81 years in the large-ROM groups. Sixty-one patients (48.41%) underwent 1-level CDA, 26 (20.63%) 2-level CDA, and 39 (30.96%) 2-level HS. The most commonly operated level with CDA was C5/6 (*n* = 75), followed by C4/5 (*n* = 36), and then C6/7 (*n* = 21). No patients underwent 2-level CDA distributed target levels in the different groups.

**Table 1:** Summary of the patient demographic data (Displayed as a number or mean ± standard deviation)

Variable	Small-ROM	Large-ROM	*P*
No. of patients, *n*	64	62	–
No. of arthroplasty levels, *n*	66	66	–
Age (range), years^a^	46.11 ± 7.87 (26–62)	43.81.58 ± 7.99 (28–63)	0.106
Sex (M/F)^c^	32/32	21/41	0.067
BMI^a^	23.67 ± 2.87	23.17 ± 3.11	0.354
T-value^a^	0.48 ± 1.16	0.45 ± 1.26	0.925
Cause^b^			0.051
Disc herniation	43	53	
Spondylosis	23	13	
Surgery type^b^			0.996
1-level CDA	31	30	
2-level CDA	14	13	
2-level HS	20	19	
Levels^b^			0.008
C4/5	12	24	
C5/6	38	37	
C6/7	16	5	
Operative time (range), min^a^	131.83 ± 36.95 (60–225)	135.29 ± 38.53 (60–300)	0.608
Blood loss (range), ml^a^	56.11 ± 33.47 (5–150)	66.69 ± 58.94 (10–350)	0.216
Follow-up (range), months^a^	35.95 ± 23.26 (18–120)	38.27 ± 23.13 (13–109)	0.587

^a^Independent t test

^b^Chi-square test

^c^Fisher exact test

### Clinical outcomes

Overall, JOA, NDI, and VAS scores showed significant post-surgical improvement (*P* < 0.001) in both the small-ROM and large-ROM groups. However, there were no significant differences in the JOA, NDI, and VAS scores between the two groups at any follow-up point (Table 2).

**Table 2:** Clinical outcomes between small-ROM and large-ROM groups

	Small-ROM	Large-ROM	*P*
JOA ^a^			
Pre-op	11.84 ± 1.48	11.85 ± 1.46	0.996
Post-op	15.92 ± 0.80	15.84 ± 0.79	0.560
NDI ^a^			
Pre-op	29.48 ± 4.99	28.15 ± 3.37	0.079
Post-op	7.58 ± 3.68	7.77 ± 3.39	0.757
VAS ^a^			
Pre-op	6.25 ± 1.41	6.29 ± 1.25	0.866
Post-op	1.39 ± 1.06	1.39 ± 0.88	0.984

^a^Independent t test

*Pre-op* Preoperatively, *Post-op* Postoperatively, *JOA* Japanese Orthopedic Association, *NDI* Neck Disability Index, *VAS* Visual Analogue Scale

### Radiographic outcomes

#### Cervical lordosis and C2-C7 ROM

**Fig. 3:**
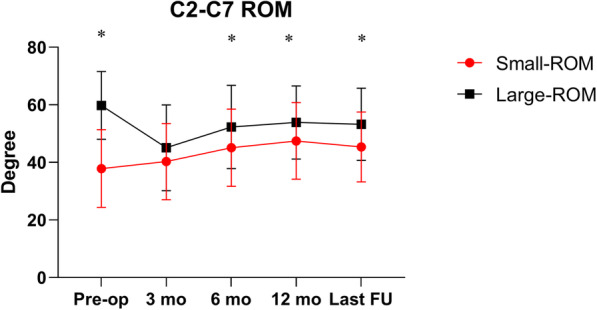
C2-C7 ROM. Patients in the small-group showed a significant increase (*P* < 0.001) in ROM, whereas those in the large-ROM group showed a significant decrease after surgery (*P* < 0.001). Asterisks (*) indicates a significant difference between the two groups (*P* < 0.05)

The preoperative cervical lordosis values (6.25° ± 11.24°) had increased significantly at the last follow-up (10.45° ± 7.90°, *P* < 0.001) for the small-ROM group, but changed slightly in the large-ROM group (12.01° ± 12.62° vs.12.06° ± 9.08°), respectively. Patients in the large-ROM group showed significantly larger cervical lordosis before surgery (*P* = 0.008), but there was no significant post-surgical difference between the two groups. The overall cervical motion increased significantly from admission to the last follow-up in the small-ROM group (37.85° ± 13.51° to 45.38° ± 12.14°, *P* < 0.001) and significantly decreased in the large-ROM group (59.79 ± 11.79° to 53.24° ± 12.56°, *P* < 0.001). The changes over the follow-up period are shown in Fig. 3.

#### Radiographic changes at the arthroplasty level

The small-ROM group showed significantly less preoperative disc lordosis than the large-ROM group (1.19° vs. 4.09°, *P* < 0.001), and tended to have less reduction (1.13° vs. 2.14°, *P* = 0.125) at the last follow-up.

**Fig. 4:**
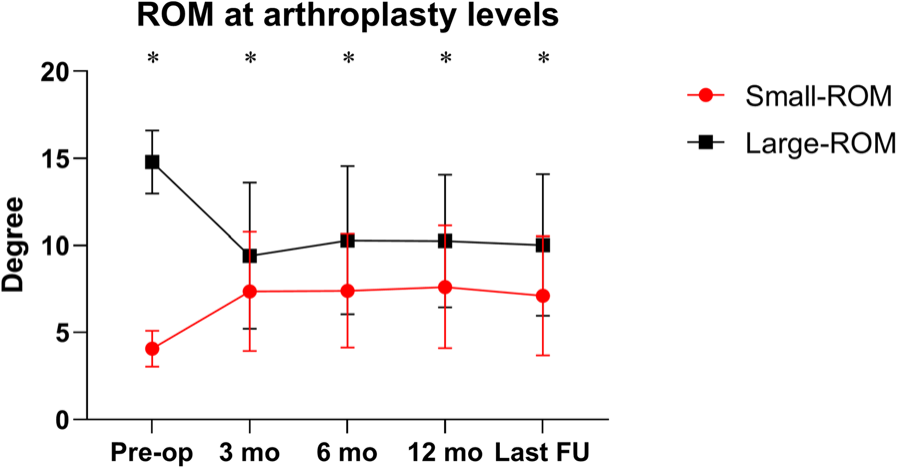
ROM at the arthroplasty levels. The index levels showed a significant increase in the small-group (*P* < 0.001), but a significant decrease in the large-ROM group after surgery (*P* < 0.001). Asterisks (*) indicates a significant difference between the two groups (*P* < 0.05)

There was a significant increase in the preoperative (4.05° ± 1.04° to 7.11° ± 3.43°) for the small-ROM group at the last follow-up, for an overall delta ROM (△ROM) of 3.05° ± 3.69° (*P* < 0.001, Fig. 4). The large-ROM group yielded an opposite trend; ROM of the arthroplasty level remarkably decreased from 14.80° ± 1.82° to 10.02° ± 4.07° with a △ROM of − 4.77° ± 4.22° (*P* < 0.001, Table 3). The segmental mobility was significantly higher in the large-ROM group, although the difference between the two groups was narrow (*P* < 0.001). The change of segmental ROM at the arthroplasty level was similar for different surgical techniques (Table 4).

**Table 3:** Radiographic outcomes between small-ROM and large-ROM groups

	Small-ROM	Large-ROM	*P*
Cervical lordosis (°) ^a^			
Pre-op	6.25 ± 11.24	12.01 ± 12.62	0.008
Post-op	10.45 ± 7.90	12.06 ± 9.08	0.288
C2-C7 ROM (°) ^a^			
Pre-op	37.85 ± 13.51	59.79 ± 11.79	< 0.001
Post-op	45.38 ± 12.14	53.24° ± 12.56°	< 0.001
△ROM	7.53 ± 15.92°	−6.55° ± 13.48°	< 0.001
Disc angle (°) ^a^			
Pre-op	1.19 ± 2.88	4.09 ± 4.09	< 0.001
Post-op	1.13 ± 3.80	2.13 ± 3.75	0.125
Segmental ROM (°) ^a^			
Pre-op	4.05 ± 1.04	14.80 ± 1.82	< 0.001
Post-op	7.11 ± 3.43	10.02 ± 4.07	< 0.001
△ROM	3.05 ± 3.69	−4.77 ± 4.22	< 0.001
Disc height (mm) ^a^			
Pre-op	5.22 ± 0.81	5.59 ± 0.85	0.012
Post-op (immediately)	6.40 ± 0.70	6.60 ± 0.77	0.368
△DH	1.18 ± 0.73	0.93 ± 0.60	0.034
ASD (%)^b^	12 (18.8%)	10 (16.1%)	0.698
HO formation (%) ^b^	40 (60.6%)	28 (42.4%)	0.037
HO classification (%)^b^			0.131
Low-grade	49 (74.2%)	56 (84.8%)	
High-grade	17 (25.8%)	10 (15.2%)	
Subsidence (%)^c^	2	3	1.000

^a^Independent t test

^b^chi-square test

^c^Fisher exact test

*Pre-op* Preoperatively, *Post-op* Postoperatively, *ROM* Range of motion, *DH* Disc height, *ASD* Adjacent segment degeneration, *HO* Heterotopic ossification

**Table 4:** Surgical type in relation to ROM in the Small-ROM and Large-ROM groups

	1-level CDA	2-level CDA	2-level HS	P
Small-ROM group				
Segmental ROM (°) ^a^				
Pre-op	4.04 ± 1.02	4.15 ± 1.08	4.00 ± 1.07	0.913
Post-op	7.25 ± 3.48	6.49 ± 3.34	7.33 ± 3.53	0.735
△ROM	3.21 ± 3.62	2.34 ± 3.61	3.33 ± 3.97	0.699
Large-ROM group				
Segmental ROM (°) ^a^				
Pre-op	14.47 ± 1.33	15.15 ± 2.06	14.99 ± 2.24	0.406
Post-op	10.18 ± 3.96	9.85 ± 4.83	9.92 ± 3.70	0.960
△ROM	−4.29 ± 3.97	−5.30 ± 4.02	−5.06 ± 4.87	0.695

^a^ One-way ANOVA

The average pre- and postoperative DH in the small-ROM group were 5.22 mm ± 0.81 mm and 6.40 mm ± 0.70 mm, respectively. The corresponding values in the large-ROM were 5.59 mm ± 0.85 mm and 6.60 mm ± 0.77 mm, respectively. There was a significant difference in DH between the two groups preoperatively (*P* = 0.012).

#### Complications

**Fig. 5:**
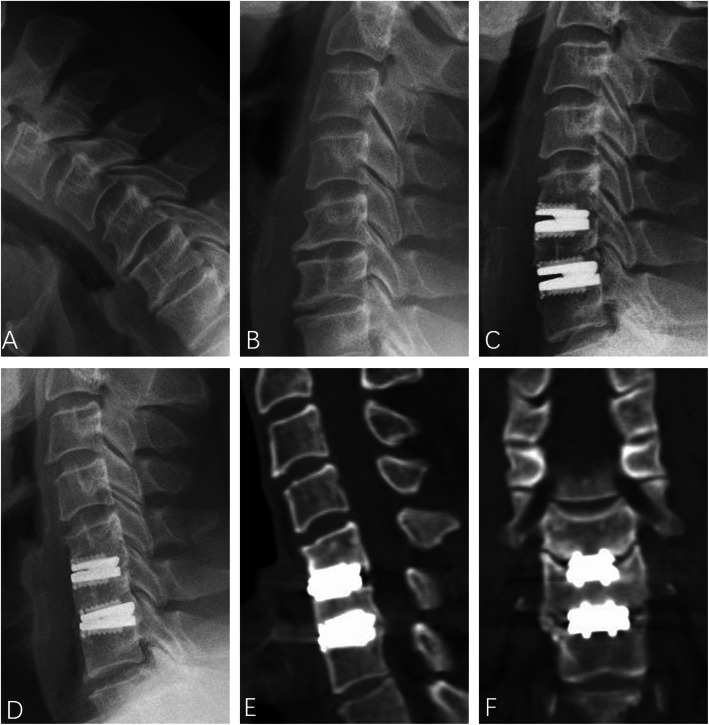
A 43-year-old male patient diagnosed with cervical spondylosis. The preoperative ROMs at C5/6 and C6/7 were 4.28° and 4.01°, respectively (**a** and **b**). The immediate postoperative X-ray (**c**) demonstrated the satisfactory location of Prestige-LP discs. The 87-month X-ray (**d**) and CT scans (**e** and **f**) showed the development of high-grade HO at C5/6 and C6/7

Twelve patients (18.8%) in the small-ROM and 10 (16.1%) in the large-ROM group had degenerative radiographic changes (Table 3). The rate of HO development was significantly higher in the small-ROM than in the large-ROM group, as determined in the last follow-up (60.6% vs. 42.4%. *p* = 0.037). Although the proportion of high-grade HO levels was higher in the small-ROM as compared to the large-ROM group, the difference was not significant (25.8% vs. 15.2%, *P* = 0.131). We divided the arthroplasty levels into positive △ROM (A) and a negative △ROM subgroups (B). Fourteen levels with less mobile (≤5°) were in subgroup B; however, 11 of them (78.6%) developed high-grade HO (Fig. 5 and Table 5). Throughout the follow-up period, two levels in the small-ROM and 3 levels in the large-ROM group occurred subsidence. No device-related complications, such as screw loosening or prosthesis migration, occurred.

**Table 5:** Subgroup analysis

	Small-ROM		P	Large-ROM		*P*
	+△ROM	-△ROM		+△ROM	-△ROM	
No. of arthroplasty levels	52	14	–	10	56	–
HO formation (%)^a^	28 (53.8%)	12 (85.7%)	0.035	3 (30%)	25 (44.6%)	0.498
HO classification (%)^a^			< 0.001			0.338
Low-grade	46 (88.5%)	3 (21.4%)		10 (100%)	46 (82.1%)	
High-grade	6 (11.5)	11 (78.6%)		0	10 (17.9%)	

^a^Fisher exact test

*HO* Heterotopic ossification

## Discussion

Many trials have substantiated the theoretical advantages of CDA over fusion in recent decades, such as preservation of motion through the segments that were operated on; however, the documented ROM of CDA shows wide variability among patients. Several investigations suggest that the preoperative ROM scale is attributed to the variability of ROM after CDA [19], but there is a shortage of data on whether limited or excessive segmental mobility should be considered as a suitable indicator for CDA. In the current study, patients were divided into the small-ROM (ROM≤5.5°) and large-ROM (> 12.5°) groups according to their preoperative index-level mobility. There was a significant difference in the distribution of operated levels between the groups, where C4/5 was more prone to hypermobility before surgery. This observation could be a reflection of the relatively spared disc disease at C4/5 compared with other segments [20, 21]. There was post-surgical relief in patients’ symptoms regardless of the preoperative segmental mobility; this may be due to the complete decompression of the spinal cord or nerve roots, disc height restoration and reconstructing stability of the cervical spine.

There was a difference in radiographic features between the small and large-ROM groups. Patients with limited segmental ROM showed significantly less global and segmental lordosis, ROM, and shorter DH. This may be due to the degenerative cascade concept; that loss of proteoglycans and water in the nucleus pulposus causes disc height loss, leading to excessive motion and instability at the early stage of disc degeneration and loss of segmental ROM at the late stage. These patients also suffered from relatively severe cervical spine degeneration. In the current study, we found that there was no significant difference in postoperative segmental ROM at the arthroplasty level among the three surgical types. The trends of segmental ROM did not alter regardless of the arthroplasty level adjacent to a fusion mass or a artificial disc. This result indicates that the change of ROM at each index level is relatively independent after surgery.

Disc with preoperatively limited ROM showed a significant increase in △ROM, by 3.05°, which parallels the observations of a previous study by Tu et al. [10]; however, unlike the current study, there were no clinical studies that had reported on CDA outcomes for discs with excessive motion. By contrast, the changes in ROM paradoxically decreased by 4.77° in discs with preoperative hypermobility. Many factors, such as overstretch of the surrounding soft tissue [21], prostheses design [10], inconsistent axis of rotation [22], and development of HO [17], could lead to decreased ROM after CDA. These findings indicate that segmental ROM could be physiologically restored by CDA using Prestige-LP discs in some cases with loss of mobility, and that the technique could partly reduce mobility in some degenerative segments with excessive motion, to achieve “dynamic” re-stability.

The key concern for patients who had excessive preoperative ROM was that the associated hypermobility would cause increased stress loading on the facet joints and accelerate their degeneration, leading to additional neck pain. However, based on the clinical and radiographic outcomes, we observed that segmental mobility preservation at the index level and the maintenance of motion through the posterior elements did not place patients at risk of increased neck pain. Thus, we proposed that selected patients with preoperatively limited or excessive segmental ROM were good candidates for CDA.

Although there is no consensus on the mechanism of HO, its development has been associated with variables such as age, sex, disc height, residual exposed endplate, and mismatch of the prosthesis [17, 23, 24]. We found that segments with preoperatively limited ROM has significantly less HO than those with excessive ROM at the last follow-up. This was in contrast to previous studies by Tu et al. [10], who reported that HO was similar for patients in the less-mobile and more-mobile groups. For further analysis, some segments in the negative △ROM subgroup were found to be more prone to severe HO, especially those with preoperatively limited ROM. One explanation could be that limit-ROM discs inherently degenerated more before surgery. Zhou et al. [25] reported that patients with more severe preoperative cervical spondylosis had higher rates of ossification formation after CDA with Bryan discs. Wu et al. [26] demonstrated that patients diagnosed with soft-disc herniation had significantly less HO (6.25%) than those diagnosed with spondylosis (58.33%). In the current study, 11 segments with preoperatively limited ROM developed HO in the negative △ROM subgroup; however, 8 of them (72.7%) had been diagnosed with cervical spondylosis before surgery. This observation may indicate that patients with preoperative cervical spondylosis are not optimal candidates for CDA if the index-level ROM is limited.

There were several limitations to the study. First, it was retrospective and carried out at a single institution, presenting inherent weaknesses and limited generalizability of the findings. Second, we evaluated the disc-levels as long as they met the inclusion criteria before surgery. However, different surgery types or disc-levels in the subaxial cervical spine may affect outcomes. The small sample size did not present adequate-subgroup data to cover all potential factors. In the current study, factors such as age, sex, and primary cause did not have any significant effect on the results, other than a tendency between the two groups, which may also attribute to the small sample size. Third, the study was limited to the Prestige-LP discs range of motion, whose FDA defined inclusion criterion of segmental ROM is 2°-20°; thus, the results may not represent any other type of prostheses. Fourth, HO formation was a time-dependent complication after CDA. The results may not have been precisely evaluated due to the extensive period of the study and the relatively small sample of long-term follow-up cases.

## Conclusions

Patients with preoperative limited segmental ROM had significantly severe postoperative HO and similar clinical improvents as patients with preoperative excessive segmental ROM. However, patients with preoperative limited segmental ROM showed increased postoperative segmental mobility, whereas patients with preoperative excessive segmental ROM paradoxically exhibited decreased postoperative segmental mobility. With proper patients selection, discs with limited or excessive mobility could benefit from CDA.

## Data Availability

Summarized data has been presented in this manuscript. The raw data is located and protected at the West China Hospital of Sichuan University. Sharing of the raw data is not recommended, because a secondary analysis is planned.

## Abbreviations

CDA: Cervical disc arthroplasty
ACDF: Anterior cervical discectomy and fusion
HS: Hybrid surgery
ASD: Adjacent segment degeneration
ROM: Range of motion
JOA: Japanese Orthopedics Association
NDI: Neck Disability Index
VAS: Visual Analogue Scale
DA: Disc angle
DH: Disc height
DDD: Degenerative disc disease
HO: Heterotopic ossification
CT: Computed tomography
MRI: Magnetic Resonance Imaging
SD: Standard deviation
